# Cancer treatment decisions for people living with dementia: Experiences of family carers, a qualitative interview study

**DOI:** 10.1111/hex.13466

**Published:** 2022-03-16

**Authors:** Catherine Hynes, Victoria J. Hodges, Lynda Wyld, Caroline Mitchell

**Affiliations:** ^1^ Academic Unit of Primary Medical Care University of Sheffield Sheffield UK; ^2^ Department of Oncology and Metabolism University of Sheffield Sheffield UK

**Keywords:** cancer, carers, comorbidity, dementia, proxy decision making, shared decision making

## Abstract

**Background:**

As the UK population ages, the prevalence of both dementia and cancer will increase. Family carers of people with dementia who are subsequently diagnosed with cancer are often involved in treatment decisions about cancer. These decisions are uniquely challenging.

**Objectives:**

To explore the experience of carers involved in cancer treatment decisions for people with dementia.

**Design:**

A cross‐sectional qualitative interview study with inductive thematic analysis.

**Setting and Participants:**

Sixteen carers of people with dementia were identified via Primary Care Research Networks and the Join Dementia Research database.

**Results:**

Three main themes were derived: ‘already at breaking point’, which describes the extreme strain that carers were already under when the cancer diagnosis was made; ‘maintaining the status quo’, which describes how despite the gravity of a cancer diagnosis, avoiding further dementia‐related deterioration was of prime importance; and ‘LPA’, which explores the benefits and frustrations of the use of lasting powers of attorney.

**Discussion:**

Current services are ill‐equipped to deal with people who have a combination of dementia and cancer. Proxy decisions about cancer care are made in the context of carer stress and exhaustion, which is exacerbated by shortcomings in service provision.

**Conclusions:**

As the prevalence of comorbid cancer and dementia rises, there is an urgent need to improve services that support carers with proxy health care decision‐making.

**Patient or Public Contribution:**

The study design was codeveloped with a local dementia‐specific patient and public involvement (PPI) group. A project‐specific PPI group was formed with support from the Alzheimer's Society Research Partnership scheme to provide further bespoke input.

## 1 INTRODUCTION

There are approximately 850,000 people living with dementia in the United Kingdom, with 95% of cases occurring in those over 65.^1^ An estimated 700,000 people, usually family members, provide informal care for someone with dementia.^2^ The prevalence of dementia in the United Kingdom is predicted to rise dramatically over coming decades as the population ages, with the greatest increase seen among those in the oldest age groups.^2^

In addition to dementia, older people are at risk of developing other age‐related comorbidities including cancer. A large English cohort study found that the odds of cancer patients also having dementia dramatically increases with age.^3^ Recent analysis of UK GP records found that 7.5% of people over 75 with cancer also have dementia.^4^ This represents a small but significant proportion of cancer patients. Dementia is associated with worse cancer outcomes across all measures, including all‐cause mortality.^5^, ^6^, ^7^ This reflects the link between dementia and comorbidities and the direct effect of dementia on survival and cancer‐specific outcomes. People with dementia are less likely to partake in screening (for breast, bowel and cervical cancer) and less likely to report early symptoms or signs of cancer, which may instead be picked up at a later stage by a carer, contributing to worse outcomes. Once diagnosed, there is evidence that patients with a diagnosis of cancer and dementia are often treated less aggressively.^8^

Any older person with a new cancer diagnosis must navigate a complex set of choices. They must balance their cancer pathology and available treatments against their own perception of their mental and physical condition, frailty and social situation and their personal views and priorities.^9^, ^10^, ^11^ People with pre‐existing dementia often do not have the capacity to make decisions of this complexity, and in these cases, where available, a family member is required to do so on their behalf.

Research examining the impact that making life‐altering decisions by proxy has on family carers is mostly limited to issues regarding dementia diagnosis and subsequent dementia care, including end‐of‐life care. Proxy decision‐making can be highly distressing, and fraught with guilt and uncertainty for carers. A lack of information and emotional support has been highlighted as a particular problem.^12^, ^13^, ^14^

The specific experience of proxy decision‐making regarding cancer treatment for people with dementia is almost wholly unexplored, yet as the prevalence of both cancer and dementia increases it is a challenge that will confront more and more people.

This study used a qualitative approach to explore the experiences of family carers who have been involved in making cancer treatment decisions on behalf of a relative living with dementia.

## 2 METHODS

### 2.1 Study design

A cross‐sectional qualitative study.

### 2.2 Participants

Family carers of people with dementia who had received a diagnosis of cancer (excluding squamous and basal cell skin cancers) within the last two years, where the carer had been required to make proxy decisions about cancer testing and treatment on the patient's behalf.

### 2.3 Recruitment

A purposive sampling approach was used to achieve a maximum variation sample.^15^ Participants were identified through two sources:

Twenty GP practices across Yorkshire and the Humber that were registered with the National Institute for Health Research (NIHR) Clinical Research Network (CRN) Portfolio performed a database search for patients coded with dementia who had received a cancer diagnosis within the last 2 years. Carers of these patients who were also registered with the practice were sent an invitation letter from the practice outlining the study. Those who returned the reply slip were contacted to arrange an interview. Due to the sensitive nature of the topic, our patient and public involvement (PPI) group felt it was important that only people who wished to take part should be known to researchers, so no information regarding details of the patients contacted by their GP practices was held by the research team. We, therefore, do not have data about the number of people who refused to take part in the study, or their reasons.

Participants were also recruited via the online NIHR database, Join Dementia Research. Four hundred and thirty volunteers who self‐identified as dementia carers living within 50 miles of Sheffield were sent an email describing the study. Those who responded and met the inclusion criteria were contacted to arrange an interview. Again, this meant data regarding participant refusal could not be collected as only a small number of those contacted would actually have been eligible.

Participants received a £15 shopping voucher to compensate them for their time. Recruitment was carried out between April 2019 and January 2020.

### 2.4 Patient and public involvement

The study design was codeveloped with a local dementia‐specific PPI group. The project idea and methodology were presented at a face‐to‐face group meeting with three people with dementia and three carers. The recruitment method was discussed in detail and they advised on the wording of the initial invitation letter that was sent. A project‐specific PPI group was formed with support from the Alzheimer's Society Research Partnership scheme to provide further bespoke input. This consists of four female former carers of people with dementia. The initial project meeting was held face‐to‐face and focused on developing the interview topic guide. Following data collection, we met virtually (due to the COVID‐19 pandemic) to discuss results and our interpretation of them.

### 2.5 Ethics and research governance

Ethics committee approval was obtained from an MHRA compliant REC (REC number 18/NW/085). Research governance approval was granted by the University of Sheffield. All participants gave written informed consent.

### 2.6 Interviews

The interview topic guide was developed with input from the research team and feedback from the project‐specific PPI group. Although no formal pilot interview was undertaken, we allowed concepts generated from initial interviews to inform and adapt the topic guide for subsequent ones.

Interviews were conducted by C. H. (a female academic GP trainee with masters‐level training and experience in qualitative research methods) between April 2019 and January 2020. A single interview took place in the carer's choice of location; either their own home, a meeting room at the University of Sheffield or a neutral, private location chosen by the carer. Interviews lasted around one hour. Only C. H. and the interviewee(s) were present, except for Interview 13 where the participant's wife (whom he cared for) was also there. Telephone or email contact was made between C. H. and the interviewee before the interview to make arrangements. In some cases, the participant's experiences were briefly discussed but the interview schedule remained he same regardless of how much information was known before the interview. Participants were aware that C. H. was a GP trainee as well as a researcher.

Interviews were audiorecorded and transcribed verbatim. Field notes were made after the interview. Participants were not asked to review the transcripts.

### 2.7 Analysis

Data were analysed inductively, using a reflexive, thematic analytical framework.^16^ Initial analysis happened alongside data collection, so emerging concepts could be explored during subsequent interviews.

Two researchers (C. H. and V. H.) conducted the initial data analysis independently, following the same process. Nvivo‐12 software^17^ was used. Transcripts were reread for familiarization and initial codes assigned to the data. C. H. and V. H. then cross‐checked their separately assigned codes together and from this developed a single set of descriptive codes. These were reviewed regularly throughout data collection with a third researcher, C. M. Once data collection was completed, C. H., V. H. and C. M. grouped the final set of codes into broader categories. The data were then rescrutinized to check for consistency and to identify outliers. further discussion developed the three overarching themes from the categories, which were reviewed and validated by the project‐specific PPI group.

## 3 RESULTS

### 3.1 Participants

Sixteen family carers were interviewed (one as a son/granddaughter diad) (Table 1, participant characteristics). Data saturation was achieved.

**Table 1:** Participant characteristics

Participant number	Sex of participant	Sex of relative living with dementia	Relationship to the person living with dementia	Age of participant	Index of multiple deprivation decile of participant (by postcode)	Ethnicity of the participant	Type of dementia their relative had	Type of cancer their relative had	Relative alive or deceased at time of interview
1	F	M	Daughter	61	6	WB	Alzheimer's	Prostate	Deceased
2^a^	F	F	Granddaughter	46	2	WB	Alzheimer's	Breast	Alive
3^a^	M	F	Son	72	2	WB	Alzheimer's/vascular	Breast	Alive
4	F	M	Daughter	48	8	WB	Alzheimer's/vascular	Bowel	Deceased
5	F	M	Daughter	–	8	WB	Alzheimer's	Bowel	Deceased
6	F	F	Daughter	–	–	WB	Alzheimer's	Breast	Alive
7	F	M	Wife	59	2	WB	Alzheimer's	Renal	Deceased
8	F	F	Daughter	37	1	WB	Lewy body	Renal	Alive
9	F	M	Wife	–	10	WB	Alzheimer's	Renal	Deceased
10	M	F	Husband	76	10	WB	Alzheimer's	Breast	Alive
11	M	F	Son	–	3	WB	Alzheimer's	Vulval	Deceased
12	F	M	Wife	–	7	WB	Frontotemporal lobe	Prostate	Alive
13	M	F	Husband	76	10	WB	Mixed	Melanoma	Alive
14	F	F	Daughter	57	4	WB	Alzheimer's	Lung	Deceased
15	F	F	Daughter‐in‐law	50	2	WB	vascular	Lung	Alive
16	F	M	Daughter	57	7	WB	Alzheimer's	Lung	Deceased

Abbreviations: F, female; M, male; WB, White British.

^a^ Participants interviewed together.

All participants had cared for patients whose dementia was sufficiently advanced by the time they were diagnosed with cancer that they were unable to make decisions about treatment independently.

A wide range of decisions relating to cancer diagnosis and treatment were covered in the interviews, from consenting for investigations and treatment (surgery, radiotherapy and chemotherapy) to palliation.

### 3.2 Themes

Three main themes were identified that describe the experience of proxy decision‐making about cancer treatment for relatives of people with dementia. These are: ‘already at breaking point’; ‘maintaining the status quo’; and ‘Lasting Powers of Attorney’ (LPA). Broadly they are framed within a person‐ and carer‐centred perspective. Together they demonstrate the stark reality of caring for a loved one with dementia: unrelenting stress, emotional turmoil and exhaustion, which is then exacerbated when additional physical comorbidity becomes an issue. Being required to make complex decisions under such circumstances creates further distress and uncertainty.

1. *Already at breaking point*
  All participants spoke of their long and difficult journeys trying to manage their relative's dementia, which began long before cancer became an issue. Many dementia carers exist at the boundaries of what they can cope with. A concept that vividly illustrates this was expressed by several participants; that a diagnosis of cancer was viewed with a sense of relief, albeit accompanied by guilt. It offered an escape from the distressing, inevitable decline of dementia and a potential release for relatives from the relentless task of caring.
*When the consultant told me it was a particularly aggressive cancer and he probably had six months to live, I remember thinking, my first thought was thank God*. P9 (female, husband had renal cancer)
*It was a way out, she'd have been dead now, no more Alzheimer's. Because my view is what people don't really appreciate: Alzheimer's is terminal, it just takes a bloody long time*. P10 (male, wife has breast cancer)
  Most participants already undertook a significant amount of caring work for their relatives at the point when cancer was diagnosed. For some, cancer caused symptoms, which, in combination with their existing dementia, further increased the care that they needed and placed additional strain on their carer:
*He started with bleeding in the urine which was distressing for him and also because if he had been thinking in his right mind, and he got a sudden urge to go to the toilet – we have the toilet down on the ground floor – he would have gone through there and then taken his things down there. But because his mind wasn't working right, he was sort of pulling his pants down halfway through the kitchen. So that was very distressing for me*. P7 (female, husband had renal cancer)
  Often the addition of a cancer diagnosis increased the burden of practical caregiving tasks, for example, attending outpatient appointments and tests and providing additional care following treatment:
*She came home, she was in her room, had to make sure she'd got drinks and food and things and she was upstairs anyway because the bathroom's closer and things like that. I did a lot of steps that week up and down the stairs! But the care, it was quite stressful because sometimes she couldn't – even though you could tell by her face that she's in pain she wouldn't tell you, or she'd complain about something totally ridiculous and really it's because she's got backache, you know, and it was quite a stressful week*. P8 (female, mother has renal cancer), following her mother's nephrectomy.
  In some cases this was literally life‐altering:
*She was due to start the radiotherapy in the August and that was when I knew I wasn't ever going to go back to work, because I thought well I can't be going to Leeds every day and try and work*. P6 (female, mother has breast cancer)
  Poor communication from healthcare professionals created additional stress for carers. This was particularly problematic during hospital admissions. Carers who were used to managing all aspects of their relative's life were suddenly excluded:
*I know that they're busy but surely you know, they could have spent a couple of minutes just talking to me, saying ‘This is what's happened’ or ‘This is where he is and this is what we're doing’ you know… And I know everybody's busy but really you need to be informed you know, your husband's not just any random person. You need to know what's going on. *P7
  Similarly, when the person with dementia was in a specialist dementia unit, staff lacked expertize in managing physical conditions, so the carers felt an additional responsibility for identifying and managing these:
*We watched him more carefully and how he was presenting pain, like checking is he scrunching his face up when he's asleep kind of thing because he's in pain. Found it really stressful for them to manage his pain in a mental health unit*. P4 (female, father had bowel cancer)
  A profound feeling of being alone in the challenges they faced was expressed by many participants. While some had good support, particularly from immediate family, others felt very isolated because no one they were close to could relate to the situation they were in:
*I've got some friends that I've known for over twenty years, so I've got quite a good support network with them, but really their parents haven't suffered with anything like that… I think unless you've had the stressful situation you can only empathise so much*. P8
*it's… making those decisions is very lonely, I've always done it on my own. And it's the same with the cancer one… supposing I've done it wrong?* P10
  Even dementia support organizations did not always have the necessary expertize to provide support to people going through such a specific situation as managing both cancer and dementia, as P10 described when he was trying to get some advice on what to expect before the best interest meeting to decide on treatment for his wife's breast cancer:
*I rang Alzheimer's Society up and said ‘have you not got anybody who's going through what I'm going to go through that could help me?’ and they said ‘no’. None*
2. *Maintaining the status quo*
  Despite the gravity of a cancer diagnosis, managing their relative's dementia and conserving their current level of functioning remained the biggest challenge and priority for carers. Cancer decisions had to be fitted around the need to minimize disruption.
*When he first said to us she'd have to go in for a biopsy… then once they'd done the biopsy on it she'd then have to go in for the (further surgery)– and I was like ‘well that's two separate operations you're putting a woman who's already got the issues she's got, you'll be putting her under major surgery twice, I know it's only keyhole but keyhole to somebody with dementia, it's massive.’* P8
  Trying to navigate a person with dementia through general NHS services provided a universal challenge that compounded carer stress. There were several instances where carers took their relatives for investigations or treatment to find that there had been no prior planning to accommodate the fact that they had dementia. Carers were left trying to contain the difficult situations, which arose as a result of inadequate preparation.
*I said ‘I'm really sorry but you need to speed up because this is a man with dementia who's not really understanding and I'm not containing him, I can see him going and if he goes the whole ward will be disrupted’, but they didn't really take heed, so they saw my dad in full force. And I was just sat there going ‘I told you.’* P5 (female, father had bowel cancer)
  Many cited the difficulties of ending up in ‘specialty silos’, where people with dementia were treated by professionals who only focused on a specialty‐specific condition (their cancer), rather than adopting a holistic approach to managing their health.
*A friend of mine who was a nurse said, ‘the surgeon will be thinking with a surgeon's head on like you know, like “Oh well if I get this out, that will sort that out”’. They are not looking at the whole thing with the whole person that's got Alzheimer's you know. So I was looking at the whole thing and him and his Alzheimer's and his other health issues. And the surgeon was only thinking I think right, we'll do this, do this and then we're sorted you know*. P7, on the decision not to have her husband go through a partial nephrectomy for renal cancer.
  Carers were left with the burden of balancing physical health care decisions against their potential impacts on dementia. They generally felt they themselves had insufficient expertize to do so and identified a lack of professional support to help them.
*In an ideal world we would have had one person who understood, like a case‐worker or somebody, that stayed with him on his journey so that it wasn't disjointed*. P4
  Some participants described occasions where doctors had made clumsy attempts to share the uncertainty and present management choices. In the context of cancer treatment decisions, this lead to confusion and increased stress:
*We saw another gentleman, which was even more confusing. He gave a whole load of information, which confused me, let alone my mum, he started – he was going off about different things and saying ‘oh you could have a massive bleed if you have the tumour removed because you've got to have some of the kidney removed’, so my mum got all confused and didn't know what was going on, what was supposed to be being done*. P8
  Particularly when considering whether or not to pursue active treatment for cancer, there were some instances where doctors had clearly decided on the best course of action, yet still made attempts to engage in ‘shared decision‐making’ with carers. This unnecessarily placed a huge burden of perceived responsibility onto the carer.
*Really that was my decision. The GP was kind of asking me that and I was having to kind of get my head round that way of thinking. Well he was suggesting that ‘yeah, she's at the end of her life and should we be, you know, trying to save her or should we just let her die? Is that alright with you? Shall we do that?’*. P3 (male, mother has breast cancer)
  Carers lacked the expertize or information to make many of the decisions that doctors attempted to share, again adding to the distress felt when trying to do the right thing for their relatives.
*We don't have the medical knowledge, you know, what we know about Alzheimer's or cancer or about epilepsy or about anything is purely through what we've gleaned from other places and you kind of feel like the professionals are saying ‘well you tell us what to do' and that's kind of not the way round you want it. You need them to have said ‘right, this is what we need to do, this will help us make this decision, so let's work it out’*. P2 (female, grandmother has breast cancer)
  Rather than empowering carers, attempts at shared decision‐making often burdened them with guilt and uncertainty, leaving them feeling responsible for a choice which, in reality, they had little influence over.
3. Lasting Powers of Attorney

A number of interviewees had LPA arrangements in place, which they strongly valued as they gave them the confidence to make decisions on their relative's behalf.

*I've never felt any guilt about that. I was always OK with it. And I really believe we acted in the best interests of him and that was the point of the Power of Attorney and Dad put that in place in I think*. P4

It could also facilitate communication with medical teams who felt more able to discuss care openly when an LPA was in place, as it allayed concerns about patient confidentiality.

*I was wanting information about that wouldn't have been released unless I'd have said ‘I've got Lasting Power of Attorney over Health' but that was kind of, like, a green light for everybody to open the… you know, just talk freely*. P4

LPAs were also a common cause of dispute between carers and health care professionals. There was often an apparent lack of knowledge and understanding within medical teams regarding the authority granted to attorneys, particularly if there was a disagreement between professionals and carers over the best course of action.

*Dad was in hospital… for the last eight days of his life and I think that all the way along the line, my authority was resisted. The view seemed to be that if he couldn't make a decision for himself then the decision would be made by the medical staff for him… I think there needs to be a lot more training within the medical profession about dealing with Attorneys and actually showing them some respect for a) what they're taking on and b) the fact that they have the power and authority to make these decisions which simply isn't accepted if it doesn't agree with what they want to do*. P1 (female, father had prostate cancer)

*We've definitely had mixed messages with that and we've ended up… we kind of learned it as we went and we ended up being able to say ‘legally I'm right and actually if you check it, you'll find you're wrong so let's just not waste time’*. P4

None of the carers had specifically discussed wishes regarding cancer treatment with their relative beforethey lost capacity. However, many used their knowledge of their relatives to predict what they would have wanted when the situation did arise. This use of substituted judgement eased the uncertainty around making proxy decisions:

*I think I was so close to my mum that I felt I could make decisions for her because we'd lived together for so long… So we'd always made decisions together over all those years. So it was a natural thing for me to make decisions on her behalf*. P14 (female, mother had lung cancer)

## 4 DISCUSSION

### 4.1 Summary

Few studies have explored cancer treatment decision‐making for patients with dementia and the experiences of family carers in this context.

The findings illustrate that relatives were often already at the limit of their capacity to cope with the burden of caring for someone with dementia before their loved one's cancer diagnosis. Decision‐making was influenced by a need to maintain their relative's well‐being and avoid provoking a deterioration in dementia‐related behaviour. For some, their situation was so challenging that the cancer diagnosis was a relief that represented a possible escape from dementia.

Services across the NHS are ill‐equipped to manage people with dementia who need to access care for other aspects of their health. Exhausted carers are left trying to contain the situations that result, causing further stress. Carers cited a lack of knowledgeable support from professionals, poor understanding of their situation and failure to view the person with dementia holistically. LPAs were viewed as supportive tools but were frequently a source of conflict with medical staff, highlighting a lack of professional knowledge in this area.

### 4.2 Strengths and weaknesses

This study is unique within the cancer‐dementia sphere because participants were recruited from community settings rather than tertiary centres. This enabled us to capture a wide range of experiences encompassing different stages of dementia and at multiple points along the cancer journey. Data saturation was achieved through a socioeconomically diverse sample across Yorkshire and the Humber. it provides an in‐depth exploration of the views of a very hard‐to‐reach group, which is underrepresented in research.^18^

This sample lacks cultural diversity; all participants were White British. This reflects the demographics of the participating CRN GP practices and also that of people who are registered with JDR. All CRN‐registered practices across Yorkshire and the Humber were invited to participate, therefore this may also reflect a lack of capacity for practices who serve more culturally diverse populations to participate in research. As a result, the transferability of results to other cultural settings is limited and the authors recognize that these findings sadly do not address the under‐representation of minority ethnic groups within dementia research.^19^ It is also acknowledged that, while people affected by dementia were instrumental in the development of this project, people with cancer were not involved in our PPI work. It is possible that some aspects of dealing with cancer specifically were not addressed in as much detail as they could have been. As discussed in Section Objectives, 2, due to the recruitment method we unfortunately do not have data regarding participant refusal or their reasons.

All interviews were carried out by the same person (a GP trainee), which is likely to have influenced the conduct of the interviews and responses of some participants (although they clearly still felt able to be critical of NHS services). This was addressed by a reflexive approach during data analysis,^20^ having a second researcher conduct an independent analysis and by triangulating findings with the research team and the project‐specific PPI group.

### 4.3 Comparison with existing literature

The challenges carers experienced when navigating their relative with dementia through the NHS is by no means a new finding. Dewing and Dijk's^21^ review concluded that people with dementia experience mostly negative consequences in general hospital settings. This was primarily due to a lack of person‐centred care and insufficient dementia knowledge and experience held by healthcare staff. Our findings support their interpretation that dementia carers also perceive their relative's experiences of hospital care negatively. Our participants raised particular issues around poor communication with hospital staff as well as lack of person‐centred care. This adds to their existing physical and emotional exhaustion.

Shared decision‐making is widely accepted as the gold standard of patient‐centred cancer care; ^22^, ^23^, ^24^ however, this study highlights the potentially negative impact it can have when presented carelessly by the clinician, an issue which many may be unaware of.^25^ Carers were often left feeling confused, or that the decision‐making responsibility was solely theirs. Participants welcomed guidance and professional opinion, as long as it was clear that their situation was carefully considered and that the person with dementia was being recognized as an individual. This supports the findings of Thorne et al.,^26^ where patients making cancer treatment decisions for themselves expressed profound gratitude when their clinician gave them a professional opinion and felt abandoned when they simply provided information and left them to make their decision alone. Similarly, Livingstone et al.^14^ found that clear advice and views from clinicians reduced guilt for carers making proxy decisions for someone with dementia.

Our participants have provided a stark picture of the reality of caring for someone with dementia in the United Kingdom, in particular when trying to manage cancer alongside dementia. Carers often feel isolated and unsupported, lacking a reliable first point‐of‐contact for advice when confronted with challenging decisions to make. In some parts of the United Kingdom Admiral nurses provide this service, but coverage is limited^27^ and few of our participants had any input from them. The 2018 NICE dementia guidelines recommend that people living with dementia should be provided with a named health or social care professional who is responsible for coordinating their care.^28^ A recent survey mapping postdiagnostic dementia services across England showed that there is a wide variation in provision, with a lack of integration and overlaps from multiple providers resulting in a confusing landscape for people with dementia and their families.^29^

LPAs are viewed as important tools for supporting proxy decision‐making; however, our findings show that they are frequently poorly understood and often challenged by medical staff. Research examining the experiences of attorneys utilizing their decision‐making authority within a UK healthcare setting is limited. Shepherd et al.^30^ examined carer experiences of using LPAs to make decisions about research participation for their relatives. In this context, many participants found that being the holder of an LPA made things much more straightforward. However, one participant explained that professional responses to the LPA varied widely, and it was not always useful. Internationally, a study in Singapore^31^ supported this; they found that the knowledge and attitudes of healthcare staff were varied. It seems that LPAs do not always provide relatives with the straightforward path to making proxy decisions that they are intended to facilitate. Further research is needed to understand the barriers to LPA use and to address this issue.

### 4.4 Implications for research and practice

People with dementia are some of our most elderly and frail patients, yet when they have to access NHS services for physical health problems they experience a system that is not equipped to provide the holistic care they need. The findings of this study highlight a need to modify cancer care pathways so that they can accommodate the needs of people with dementia, from initial investigations and diagnosis all the way through to treatment and palliative care. Codesign of services with people with dementia and carers has the potential to result in services that better meet their needs.

Clear routes to support for carers are needed from the point of diagnosis throughout the disease process. There is a strong argument for such services being community‐based rather than in secondary care, where they are better placed to target the needs of their specific populations.^32^ However community services must be adequately and sustainably resourced and funded to provide the quality of support that people living with dementia and their carers so desperately need.

This study found that LPAs for health and welfare currently do not empower relatives to make decisions in the way they are intended. In fact, they were frequently a source of conflict between carers and HCPs. Carers were left feeling they had to fight for the care that they and their relative wanted, when their forward planning in preparing the LPA was intended to make such situations more straightforward. LPAs have existed in the United Kingdom since 2007 as part of the Mental Capacity Act.^33^ Research to understand the barriers and facilitators to their appropriate use in health settings may be useful.

## 5 CONCLUSION

The prevalence of comorbid cancer and dementia will continue to rise. Decisions about cancer care for people with dementia are complex and carers making these decisions by proxy must balance competing priorities in stressful and emotive circumstances. Services are currently ill‐prepared to support them in this, exacerbating their distress.

## CONFLICTS OF INTEREST

The authors declare no conflicts of interest.

## Supporting information

Supporting information.

## Data Availability

The data that support the findings of this study are available from the corresponding author upon reasonable request.
