# Supplementary material for: Cancer treatment decisions for people living with dementia: Experiences of family carers, a qualitative interview study
Source: Health Expect. 2022 Mar 16;25(3):1131–9. doi: 10.1111/hex.13466 (PMC9122422; doi:10.1111/hex.13466)
Supplement: Supplementary file 1 — Supporting information. [file HEX-25--s001.docx]

**COREQ (COnsolidated criteria for REporting Qualitative research) Checklist**

**Domain 1: Research team and reflexivity**

| 1. | Interviewer/facilitator | Which author/s conducted the interview or focus group? | CH |
| --- | --- | --- | --- |
| 2. | Credentials | What were the researcher's credentials? *E.g. PhD, MD* | CH: MbChB, MPH  VH: MbChB, MRCGP  LW: PhD, FRCS  CM: MD, FRCGP |
| 3. | Occupation | What was their occupation at the time of the study? | CH: Academic GP trainee  VH: Clinical Academic GP  LW: Professor of Surgery, Consultant surgeon  CM: GP, Senior Clinical Lecturer |
| 4. | Gender | Was the researcher male or female? | All female |
| 5. | Experience and training | What experience or training did the researcher have? | CH: Masters level experience and training in conducting qualitative research  VH: Prior experience of conducting qualitative research; in-house training on research methods via University department  LW and CM both extensive research experience |

Personal Characteristics

Relationship with participants

| 6. | Relationship established | Was a relationship established prior to study commencement? | In some cases a brief conversation by phone for the purposes of arranging an interview – sometimes led to a brief chat about the participant’s situation/experience but this did not affect the interview structure |
| --- | --- | --- | --- |
| 7. | Participant knowledge of the interviewer | What did the participants know about the researcher? e*.g. personal goals, reasons for doing the research* | Participants were aware that CH was a GP trainee and researcher – discussed prior to consent process |
| 8. | Interviewer characteristics | What characteristics were reported about the interviewer/facilitator? e.g. *Bias, assumptions, reasons and interests in the research topic* | Being a GP trainee may have influenced conduct of interviews and responses.  See paper for commentary on using reflective field notes and reflexivity both as individuals and within our group discussions and the limitations of our biomedical backgrounds |

**Domain 2: Study design**

Theoretical frameworks

| 9. | Methodological orientation and Theory | What methodological orientation was stated to underpin the study? *e.g. grounded theory, discourse analysis, ethnography, phenomenology, content analysis* | Reflexive thematic analysis |
| --- | --- | --- | --- |

Participant selection

| 10. | Sampling | How were participants selected? *e.g. purposive, convenience, consecutive, snowball* | Purposive |
| --- | --- | --- | --- |
| 11. | Method of approach | How were participants approached? e*.g. face-to-face, telephone, mail, email* | GP practice communication (letters) and by email (JDR) |
| 12. | Sample size | How many participants were in the study? | 16 |
| 13. | Non-participation | How many people refused to participate or dropped out? Reasons? | Not known |

Setting

| 14. | Setting of data collection | Where was the data collected? e*.g. home, clinic, workplace* | Home/Academic Unit/neutral location of participant’s choosing |
| --- | --- | --- | --- |
| 15. | Presence of non-participants | Was anyone else present besides the participants and researchers? | No |
| 16. | Description of sample | What are the important characteristics of the sample? *e.g. demographic data, date* | Relationship to person with dementia; type of cancer and dementia their relative had; gender; age |

Data collection

| 17. | Interview guide | Were questions, prompts, guides provided by the authors? Was it pilot tested? | Interview topic guide used (attached) however this was allowed to develop during data collection to explore emerging themes; not pilot tested |
| --- | --- | --- | --- |
| 18. | Repeat interviews | Were repeat interviews carried out? If yes, how many? | No |
| 19. | Audio/visual recording | Did the research use audio or visual recording to collect the data? | Interviews were audiorecorded |
| 20. | Field notes | Were field notes made during and/or after the interview or focus group? | Yes |
| 21. | Duration | What was the duration of the interviews or focus group? | About 1 hour |
| 22. | Data saturation | Was data saturation discussed? | Yes in regular analysis meetings |
| 23. | Transcripts returned | Were transcripts returned to participants for comment and/or correction? | No |

**Domain 3: Analysis and findings**

Data analysis

| 24. | Number of data coders | How many data coders coded the data? | 3 |
| --- | --- | --- | --- |
| 25. | Description of the coding tree | Did authors provide a description of the coding tree? | No |
| 26. | Derivation of themes | Were themes identified in advance or derived from the data? | Inductively |
| 27. | Software | What software, if applicable, was used to manage the data? | Nvivo 12 |
| 28. | Participant checking | Did participants provide feedback on the findings? | No, but PPI group did |

Reporting

| 29. | Quotations presented | Were participant quotations presented to illustrate the themes / findings? Was each quotation identified? e*.g. participant number* | Yes |
| --- | --- | --- | --- |
| 30. | Data and findings consistent | Was there consistency between the data presented and the findings? | Yes |
| 31. | Clarity of major themes | Were major themes clearly presented in the findings? | Yes |
| 32. | Clarity of minor themes | Is there a description of diverse cases or discussion of minor themes? | Yes |

*Allison Tong, Peter Sainsbury, Jonathan Craig, Consolidated criteria for reporting qualitative research (COREQ): a 32-item checklist for interviews and focus groups, International Journal for Quality in Health Care, Volume 19, Issue 6, December 2007, Pages 349–357,*[*https://doi.org/10.1093/intqhc/mzm042*](https://doi.org/10.1093/intqhc/mzm042)
